# Using preoperative control nutritional status scores as prognostic factors for endometrial cancer

**DOI:** 10.3389/fonc.2023.1126576

**Published:** 2023-04-26

**Authors:** Jing Yuan, Qing Wang, Jiumei Cheng, JinJuan Wang, Ying Zhang

**Affiliations:** Gynecological Mini-Invasive Center, Beijing Obstetrics and Gynecology Hospital, Capital Medical University, Beijing Maternal and Child Health Care Hospital, Beijing, China

**Keywords:** endometrial carcinoma, control nutritional status, surgical excision, overall survival, risk factors, prognostic ratios

## Abstract

**Background:**

Previous investigations have reported that controlling nutritional (CONUT) status scores, incorporating total cholesterol (TC) and serum albumin (SA) values, and total lymphocyte (LY) counts, are reliable malignant tumor predictors. However, CONUT scores for predicting endometrial cancer (EC) remain unexplored.

**Objective:**

To evaluate preoperative CONUT scores as prognostic factors for postoperative EC.

**Methods:**

We retrospectively evaluated preoperative CONUT scores in 785 surgically resected EC patients at our hospital between June 2012 and May 2016. Using time-dependent receiver operating characteristic (ROC) analyses, patients were split into: 1) CONUT-high (CH) (≥1) and 2) CONUT-low (CL) (<1) groups. Relationships between CONUT scores and different clinicopathological, pathological differentiation, muscle layer infiltration depth, and prognosis factors were examined, and Cox regression analyses performed to assess prognostic values on overall survival (OS) rates.

**Results:**

We assigned 404 (51.5%) and 381 (58.5%) patients to CH and CL groups, respectively. In the CH group, body mass index (BMI), prognostic nutrition index (PNI), and LY/monocyte ratios (LMR) were decreased, however, neutrophil/LY (NLR) and platelet/LY ratios (PLR) were increased. Pathological differentiation analyses showed that G1 proportions were higher in the CL group, while G2 and G3 proportions were more prevalent in the CH group. Muscle layer infiltration depth in CL patients was < 50%, while that it was ≥50% in the CH group. No significant differences in OS rates were recorded between CH and CL groups over 60 months. However long-term survival (LTS) rates after 60 months in the CH group were significantly lower when compared with the CL group, and was more obvious in type II EC patients. Also, periuterine infiltration and preoperative CONUT scores were independent prognostic factors for OS rates as indicated by multi-factor analyses.

**Conclusion:**

CONUT scores not only facilitated the estimation of nutritional status, but were highly beneficial for predicting OS rates in patients with EC after curative resection. CONUT scores provided high predictive values for LTS rates over 60 months in these patients.

## Introduction

In the female reproductive system, endometrial cancer (EC) is one of the most common malignant tumors, with an increasing global incidence in younger females (1). According to 2019 China Cancer Center data, EC incidence rates in China were 10.3/100,000 and mortality rates were 1.9/100,000 (2). The main EC treatment is surgical excision, while radiotherapy and/or chemotherapy are common adjuvant treatments.

Several factors impact EC prognosis outcomes. Recent studies suggested that nutrition and immune inflammation were closely associated with tumor occurrence and development, and crucial for patient survival and prognosis (3, 4). Controlling nutritional (CONUT) status scores was proposed by de Ulíbarri in 2005 (5). It encompass three main indicators: total cholesterol (TC), serum albumin (SA), and lymphocyte (LY) counts, and represent bodily nutritional status, immune function, and lipid metabolism. CONUT was initially identified as a tool for the early detection and continuous control of hospital under-nutrition. Later, CONUT scores were shown to be closely related to cervical, lung, cholangiocarcinoma, and other malignant cancer prognoses (5–7).

However, CONUT scores and their impact on EC prognosis outcomes are poorly understood. Therefore, we evaluated CONUT scores and other immune nutritional indicators (e.g., prognostic nutrition index (PNI), neutrophil/LY ratio (NLR), LY to monocyte ratio (LMR), and platelet/LY ratio (PLR) to clinically evaluate EC prognosis outcomes.

## Materials and methods

### Background

We retrospectively analyzed clinicopathological data from patients with EC who had undergone initial surgery and pathological staging at our hospital between June 2012 and May 2016.

Inclusion criteria were: 1) Radical-intent resection and postoperative pathology confirming EC had been performed; 2) No adjuvant therapy administration pre-surgery; 3) Patients having complete clinicopathological data; 4) Patients aged > 25 years old at diagnosis, and 5) All follow-up data.

Exclusion criteria were: 1) Before EC surgery, patients with liver cirrhosis, hepatitis or other serious liver diseases, severe infection, kidney diseases, cardiovascular and cerebrovascular diseases or blood system diseases; 2) EC combined with other malignancies; 3) Pathologically confirmed EC after hysterectomy for other reasons (e.g., hysteromyoma, adenomyosis, and endometrial atypical hyperplasia); and 4) Death within 1 month post-surgery.

This study was approved by the Human Ethics Committees of Beijing Obstetrics and Gynecology Hospital, Capital Medical University (No. 2022-KY-037-01).

### Study variables

All patients underwent surgery-staging assessments for EC, with operations performed by experienced surgical teams. Postoperative treatments were provided according to national guidelines. After treatments, regular outpatient reviews and follow-up telephone calls were conducted.

All clinicopathological data were gathered using the medical records system. Data included age, height, weight, chronic history, menstruation, and other surgical and pathological details, including histological type, tissue differentiation, peritoneal lavage fluid, lesion infiltration area, and lymph node status. Surgical pathological staging was determined using International Federation of Gynecology and Obstetrics (FIGO) guidelines (2009). EC was classified into endometrioid (type I) and nonendometrioid (type II, mainly serous) subtypes using histology.

We collected blood samples 7 days before surgery to assess total peripheral blood LY counts, TC levels, SA levels, platelets (PLTs), neutrophils, monocytes, and other biochemical, coagulation, and tumor marker information.

### CONUT scores and other scoring systems

CONUT scores were calculated as indicated in **Table 1**. Total LY counts, SA, and TC levels in peripheral blood were categorized into quartiles and assigned scores. Total CONUT scores ranged from 0–12; a higher score indicated a worse nutritional status.

**Table 1:** Nutritional assessments using the CONUT scoring system.

Parameters	Normal	Light	Moderate	Severe
Serum albumin (g/dL)	≥3.5	3.0-3.49	2.5-2.9	<2.5
Score	0	2	4	6
Total lymphocyte (count/mm^3^)	≥1600	1200-1599	800-1199	<800
Score	0	1	2	3
Total cholesterol (mg/dl)	≥180	140-179	100-139	<100
Score	0	1	2	3
Total score	0-1	2-4	5-8	9-12

From peripheral blood, the PNI was calculated as 10 × albumin concentration (g/dl) + 0.005 × total LY counts.

The NLR reflected the absolute neutrophil count divided by the absolute LY count.

The PLT to LY ratio (PLR) was the PLT count divided by the absolute LY count.

The LMR was based on the LY count divided by the monocyte count.

### Determining cut-off values

To determine optimal cut-off values, we used receiver operating characteristic (ROC) curves and the Youden index. The optimal preoperative CONUT cut-off value was 1. As indicated, patients were assigned to CONUT-high (CH) (≥1; n=404) and CONUT-low (CL) (<1; n=381) groups.

Also, optimal PNI (52.83), NLR (1.9), PLR (175), LMR (6.45), and age (60.5 years) cut-off values were generated using ROC curve analyses and classified.

The body mass index (BMI) cutoff value was 20 kg/m^2^ (6). Carcinoembryonic antigen (CEA) (5 ng/ml), cancer antigen (CA) 199 (27 ng/ml), and CA125 (35 U/ml) cutoff values were indicated by assay instructions.

### Follow-up

We followed-up 785 patients for 60 months; the final follow-up deadline was December 31^st^, 2021 and 767 patients completed the final follow-up (18 were lost). The overall survival (OS) rate was considered the time from diagnosis to death or the last follow-up.

Patients were followed-up every 3–6 months over the 2 years after the operation, then every 6 months over 3 years, and then every year thereafter.

At each follow-up visit, patients were asked about their symptoms (e.g., vaginal bleeding, abdominal pain, etc.) and had a physical exam. They had an ultrasound every 6–12 months. When recurrence was suspected, Computed Tomography (CT), Magnetic Resonance Imaging (MRI), or Positron Emission Tomography-Computed Tomography (PET-CT) examinations were performed.

### Statistical analyses

Chi-square or Fisher’s exact tests were used to analyze categorical variables, which were represented by numbers (%). Optimal CONUT cut-off scores were determined by ROC curves and the Youden index, with patients assigned to CH and CL groups. Spearman’s correlation analyses were used to examine correlations between clinicopathological parameters and CONUT scores. OS rates were analyzed using the Kaplan-Meier method and compared using Log-Rank (Mantel-Cox) tests. We performed univariate and multivariate Cox proportional hazard regression analyses on all variables to determine independent EC prognostic factors. SPSS Software (Ver. 20.0) was used for all analyses and P<0.05 values were statistically significant.

## Results

### Determining optimal CONUT cut‐off scores

**Figure 1:**
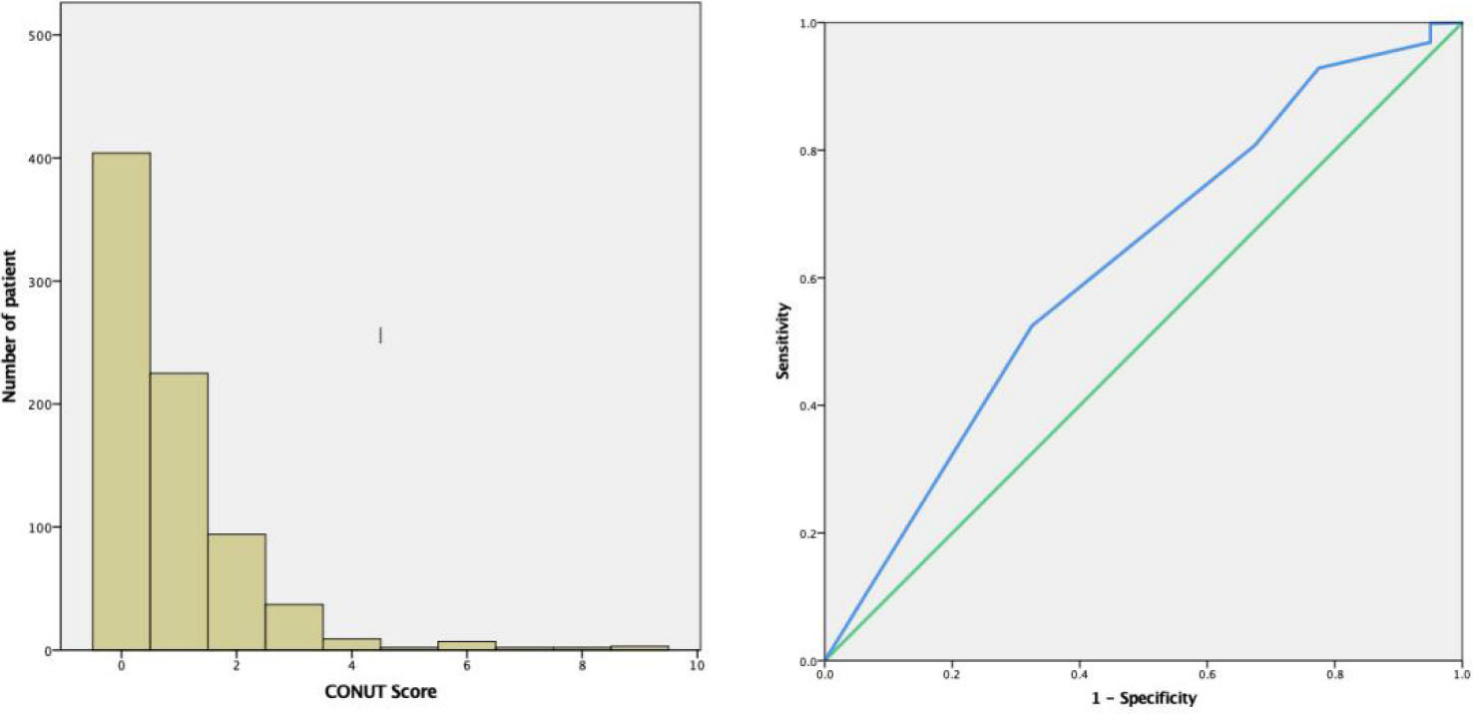
Time-related ROC curves showing preoperative CONUT scores for predicting 60 month overall survival (OS) rates. The optimal CONUT cut-off score was 1. (AUC=0.618, 95% confidence interval (CI): 0.53–0.71, P=0.01. Specificity = 67.5% and Sensitivity = 51.7%). ROC, receiver operating characteristic; AUC, area under curve; CONUT, controlling nutritional status.

Based on preoperative CONUT scores from 785 patients, cut-off values were determined using ROC curves, with patients divided into CH (≥1, n=381) and CL groups (<1, n=404) (**Figure 1**).

### Correlations between clinical indicators and CONUT scores

These were indicated by Spearman’s correlation analyses; CONUT scores were significantly positively associated with prothrombin time (PT), thrombin time (TT), international normalized ratio (INR) in EC patients (P<0.05), while significant negative correlations were identified with BMI, albumin, triglyceride, high-density lipoprotein, low-density lipoprotein, TC, and LY counts (P<0.05) (**Table 2**).

**Table 2:** CONUT scores and some clinical indicators show positive and negative correlations.

N=785	BMI	PT	TT	INR	ALB	TG	HDL	LDL	TC	LY
R	-0.07	0.21	0.11	0.10	-0.22	-0.01	-0.13	-0.39	-0.48	-0.53
P value	0.04	0.00	0.00	0.00	0.00	0.00	0.00	0.00	0.00	0.00

R, correlation coefficient; BMI, body mass index; PT, prothrombin time; TT, thrombin time; INR, international normalized ratio; ALB, albumin; TG, triglyceride; HDL, high-density lipoprotein; LDL, low-density lipoprotein; TC, cholesterol; LY, lymphocyte count.

### Correlations between clinicopathological factors and CONUT scores

In our cohort, 381 (48.5%) and 404 patients (51.5%) were in CH and CL groups, respectively. The median age was 54 years old, with a median follow-up of 83 months. BMI, PNI, and LMR were reduced, and NLR and PLR elevated in the CH group (P<0.05). Tumor pathological differentiation indicated that G1 was more prevalent in CL patients, while G2 and G3 were more prevalent in CH patients (P<0.05). Muscle layer infiltration depth in CL patients was < 50%, while it was ≥50% in CH patients (**Table 3**).

**Table 3:** CONUT scores and some clinicopathological factors are correlated.

	Total	CL group, n (%)	CH group, n (%)	χ²	P value
Age				0.08	0.78
<60.5	607 (77.3%)	314 (77.72%)	293 (76.90%)		
≥60.5	178 (22.7%)	90 (22.28%)	88 (23.10%)		
BMI (kg/m^2^)				8.07	**0.00**
<20.0	33 (4.2%)	9 (2.23%)	24 (6.30%)		
≥20.0	752 (95.8%)	395 (97.77%)	357 (93.70%)		
Menopause				1.09	0.30
no	319 (40.6%)	157 (38.86%)	162 (42.52%)		
yes	466 (59.4%)	247 (61.14%)	219 (57.48%)		
Relapse				1.02	0.31
no	774 (98.6%)	400 (99.01%)	374 (98.16%)		
yes	11 (1.4%)	4 (0.99%)	7 (1.84%)		
PNI				87.73	**0.00**
<52.83	478 (60.9%)	182 (45.05%)	296 (77.69%)		
≥52.8	307 (39.1%)	222 (54.95%)	85 (22.31%)		
NLR				44.03	**0.00**
<1.9	299 (38.1%)	199 (49.26%)	100 (26.25%)		
≥1.9	486 (61.9%)	205 (50.74%)	281 (73.75%)		
PLR				93.59	0.00
<175.0	627 (79.9%)	377 (93.32%)	250 (65.62%)		
≥175.0	158 (20.1%)	27 (6.68%)	131 (34.38%)		
LMR				49.36	**0.00**
<6.45	452 (57.6%)	184 (45.54%)	268 (70.34%)		
≥6.45	333 (42.4%)	220 (54.46%)	113 (29.66%)		
CA199 (U/ml)				0.48	0.82
<27	351 (67.5%)	175 (67.05%)	176 (67.95%)		
≥27	169 (32.5%)	86 (32.95%)	83 (32.05%)		
CA125 (U/ml)				1.52	0.22
<35	516 (85.7%)	271 (87.42%)	245 (83.90%)		
≥35	86 (14.3%)	39 (12.58%)	47 (16.10%)		
CEA (ng/L)				0.71	0.40
<5	513 (98.8%)	260 (99.24%)	253 (98.44%)		
≥5	6 (1.2%)	2 (0.76%)	4 (1.56%)		
Pathological classification				0.26	0.61
type I	719 (91.6%)	372 (92.08%)	347 (91.08%)		
type II	66 (8.4%)	32 (7.92%)	34 (8.92%)		
Surgical staging				6.63	0.09
I	607 (77.3%)	321 (79.45%)	286 (75.07%)		
II	98 (12.5%)	46 (11.39%)	52 (13.65%)		
III	63 (8.0%)	33 (8.17%)	30 (7.87%)		
IV	17 (2.2%)	4 (0.99%)	13 (3.41%)		
Degree of differentiation				7.46	**0.02**
G1	437 (55.7%)	243 (60.15%)	194 (50.92%)		
G2	263 (33.5%)	125 (30.94%)	138 (36.22%)		
G3	85 (10.8%)	36 (8.91%)	49 (12.86%)		
Lymphatic vascular space infiltration				1.69	0.19
no	613 (78.1%)	323 (79.95%)	290 (76.12%)		
yes	172 (21.9%)	81 (20.05%)	91 (23.88%)		
Parastatal infiltration				1.57	0.21
no	753 (95.9%)	391 (96.78%)	362 (95.01%)		
yes	32 (4.1%)	13 (3.22%)	19 (4.99%)		
Infiltration of muscle layer				5.99	**0.01**
< 1/2	585 (74.5%)	316 (78.22%)	269 (70.60%)		
≥1/2	200 (25.5%)	88 (21.78%)	112 (29.40%)		
Cervical interstitial infiltration				1.33	0.25
no	673	352(87.13%)	321 (84.25%)		
yes	112	52 (12.87%)	60 (15.75%)		
Aortic lymph node metastasis				0.15	0.70
no	772 (98.3%)	398 (98.51%)	374 (98.16%)		
yes	13 (1.7%)	6 (1.49%)	7 (1.84%)		
Pelvic lymph node metastasis				0.34	0.85
no	747 (95.2%)	385 (95.30%)	362 (95.01%)		
yes	38 (4.8%)	19 (4.70%)	19 (4.99%)		

CH, CONUT-high; CL, CONUT-low; BMI, body mass index; PNI, prognostic nutrition index; NLR, neutrophil/lymphocyte ratio; PLR=platelet/lymphocyte ratio; LMR, lymphocyte to monocyte ratio; CA199, cancer antigen 199; CA125, cancer antigen 125; CEA, Carcinoembryonic Antigen. Bold and highlighted for significant p-values.

### CONUT score associations with OS rates

All patients (785) were followed-up for 60 months. At final follow-up, 18 were lost and 767 remained. Of these, OS rates were lower in CH patients when compared with CL patients (91.52% vs. 95.62%). Further subgroup analyses showed that type I patients (704 cases, 91.79%) with high CONUT scores had lower OS rates when compared with low-scoring CONUT patients (95.77% vs. 96.66%). The same trend was identified in type II patients (75.76% vs. 83.33%).

**Figure 2:**
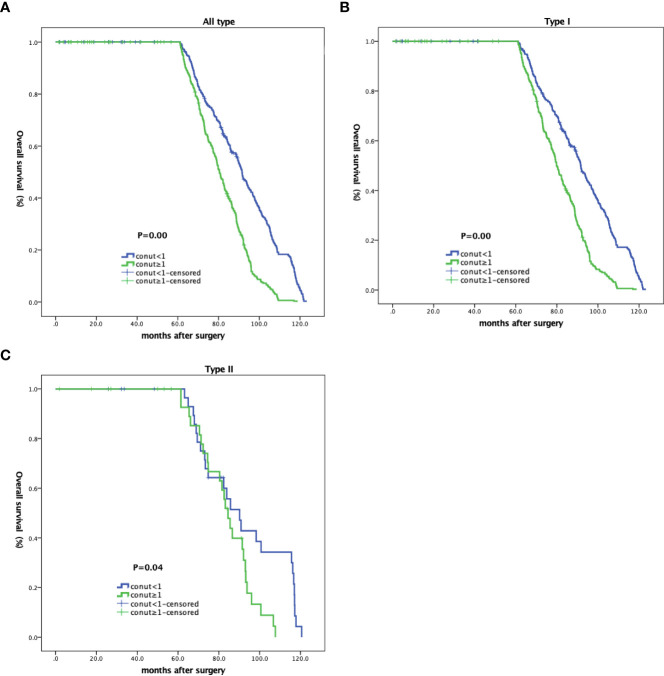
Kaplan-Meier curves showing overall survival in EC patients based on CONUT scores. **(A)** All patients; **(B)** type I; and **(C)** type II patients.

As shown (**Figure 2**), no significant differences in OS rates were observed between CH and CL groups over 60 months. LTS rates after this time in the CH group were significantly lower when compared with the CL group, and was more obvious in patients with type II EC (**Table 4** and **Figure 2**).

**Table 4:** CONUT score associations with overall survival (OS) rates.

N=767		CONUT groups and OS rates (%)		Log Rank (Mantel-Cox)	P value
		CL (%)	CH (%)		
OS rate		389 (95.62%)	378 (91.52%)	89.99	0.00
Pathological type	type I	359 (96.66%)	345 (95.77%)	86.490	0.00
	type II	30 (83.33%)	33 (75.76%)	3.923	0.04
	total			90.10	0.00

CH, CONUT-high; CL, CONUT-low; OS, Overall survival.

### Analyzing prognostic factors for OS rates

Univariate Cox regression investigations indicated that NLR, PLR, CONUT groups, and periuterine infiltration were correlated with OS rates (P<0.05). Multivariate regression analyses showed that the risk of death in EC patients without periuterine invasion was 0.56 times when compared with patients with periuterine invasion. The death risk in patients with PNI<52.83 was 1.23 times higher than PNI≥52.83, with PLR≥175.0 approximately1.36 times higher than PLR<175.0. LMR<6.45 was 1.24 times higher than LMR≥6.45, while the death risk in patients with CONUT scores ≥1 was 1.22 times higher when compared with patients with CONUT scores <1. CONUT scores, PLR, LMR, PNI, and periuterine invasion were independent prognostic factors for OS (**Table 5**).

**Table 5:** Univariate and multivariate Cox regression analyses of EC prognostic factors.

	Univariate HR (95% CI)	P value	Multivariate HR (95% CI)	P value
Age	0.850 (0.707, 1.022)	0.083	1.206 (0.982, 1.481)	0.074
BMI	0.987 (0.839, 1.162)	0.878	1.036 (0.878, 1.223)	0.672
Menopause	1.023 (0.882, 1.187)	0.763	1.010 (0.852, 1.198)	0.905
Relapse	0.890 (0.398, 1.989)	0.776	0.947 (0.420, 2.132)	0.895
CONUT	2.125 (1.812, 2.491)	**0.00**	1.216 (1.150, 1.285)	**0.000**
PNI	1.048 (0.902, 1.218)	0.542	1.232 (1.044, 1.455)	0.014
NLR	1.163(1.000, 1.352)	**0.049**	1.170 (0.989, 1.384)	0.067
PLR	1.475 (1.225, 1.776)	**0.00**	1.355 (1.100, 1.669)	**0.004**
LMR	1.088 (0.938, 1.261)	0.265	1.240 (1.052, 1.462)	**0.010**
Pathological classification	1.031 (0.773, 1.375)	0.836	1.144 (0.829, 1.580)	0.413
Surgical staging	0.900 (0.799, 1.013)	0.080	0.929 (0.759, 1.137)	0.475
Degree of differentiation	1.078 (0.964, 1.205)	0.187	1.122 (0.970, 1.298)	0.121
Lymphatic vascular space infiltration	0.936 (0.778, 1.127)	0.487	0.981 (0.779, 1.234)	0.869
Parastatal infiltration	0.609 (0.390, 0.950)	**0.029**	0.556 (0.324, 0.955)	**0.033**
Infiltration of muscle layer	0.885 (0.743, 1.054)	0.170	0.844 (0.693, 1.029)	0.093
Cervical interstitial infiltration	0.965 (0.812, 1.147)	0.686	1.058 (0.867, 1.290)	0.580
Aortic lymph node metastasis	1.556 (0.651, 3.720)	0.320	2.521 (0.923, 6.889)	0.071
Pelvic lymph node metastasis	0.798 (0.512, 1.244)	0.320	0.930 (0.518, 1.670)	0.808

HR, Hazard ratio; CI, confidence interval; NLR, neutrophil/lymphocyte ratio; PLR, platelet/lymphocyte ratio; CONUT, controlling nutritional status. Bold and highlighted for significant p-values.

## Discussion

Our study indicated that preoperative CONUT scores were independent prognostic factors for OS, especially for long-term survival > 60 months, in patients with EC. Similar to PLR, LMR, and PNI, scores, it had independent predictive values for EC OS rates. As a method evaluating immune nutritional status in patients, CONUT scores can predict prognoses in patients with multiple solid tumors (7–11). Importantly, our study is the first to determine the prognostic significance of CONUT scores for EC and shows these scores were correlated with BMI, PNI, LMR, NLR, and PLR scores. Patients with high CONUT scores had poor tumor differentiation (G2 and G3 were the more common, P<0.05) and deep myometrial invasion (> 1/2 depth, P<0.05). High CONUT scores were significantly associated with poor OS rates (low 95.62% vs. high 91.52%, P<0.001).

Recently, considerable research has focused on interactions between inflammation and malignant tumors (4). NLR, PLR, and LMR are systemic inflammatory indicators, which are generated by neutrophil and LY, PLT and LY, and LY and monocyte ratios, respectively. Previous studies reported that NLR, PLR, and LMR values had predictive significance for breast, bladder, lung, ovarian, endometrial, cervical, hepatocellular, and other cancers (12–20).

LYs are involved in cell-related anti-tumor immune responses. Increased LY infiltration is related to improved outcomes in patients with breast and colorectal cancer (21, 22). In patients with high NLR or PLR, LY population percentages are relatively low and patient prognoses are poor (20, 23). In patients with higher LMR, LY population percentages are relatively high and patient prognoses are better. Our univariate Cox regression investigations indicated that NLR and PLR levels correlated with OS rates in patients with EC (P<0.05), while in multivariate regression analyses, only PLR was significantly correlated with OS rates in patients (P<0.01). Patients with PLR≥175.0 had a 1.355 times higher risk of death when compared with patients with PLR<175.0.

In recent years, associations between impaired nutritional status and poor prognoses in patients with malignant tumors have received considerable research attention (24). Albumin is generated in the liver and is the most abundant plasma protein. SA is an important factor used to evaluate patient nutritional status. Hypoalbuminemia indicates nutritional decline in patients with severe disease, and also malnutrition in cancer patients, and is reportedly associated with poor prognoses, increased staging, and reduced OS rates due to malignant tumors (25–28). Preoperative hypoproteinemia patients have also been shown to have increased tumor spread rates and increased risks from adverse outcomes within 1 month after surgery (25). SA levels before treatment are independent prognostic parameters for disease-free and progression-free survival in EC patients (29). Hypoalbuminemia is related to reduced OS rates in patients with EC and/or ovarian cancer (26, 27), and also with increased hepatocellular carcinoma invasiveness (28).

The PNI is a comprehensive indicator combining nutritional and immune status, and is a linear prediction model based on preoperative SA levels and total LY counts. Also, PNI is an independent prognostic factor in EC; patients with a PNI≥45 before surgery have a 45% lower risk of overall mortality and cancer-specific mortality risk when compared with patients having a PNI<45 (30). Other investigations confirmed that low PNI values were associated with adverse outcomes in malignant ovarian, cervical, liver, lung, colon, and pancreatic cancers (34–40). In our study, univariate Cox regression analyses did not identify a correlation between PNI and OS rates in EC, while multivariate regression analyses showed that patients with low PNI values had higher mortality rates when compared with patients with high PNI values (P<0.05).

CONUT scores are relatively new immune nutrition indicators, and include TC serum levels based on PNI values. Cholesterol is a vital lipid with roles in cell membrane formation and maintains many cellular and bodily activities. Hypocholesterolemia affects cell membrane fluidity, reduces cell surface receptor migration and transmembrane signal transmission, and affects several key biochemical pathways. TC levels are reportedly related to tumor progress and patient survival. Previously, a 19-year prospective investigation followed 172,210 patients (41) and reported that low TC serum levels had distinct short-term correlations with high cancer incidence rates, but no long-term correlations were identified.

When CONUT scores are combined with LY counts, TC, and SA levels, they comprehensively reflect patient nutritional and immune status; those with high CONUT scores have poor nutritional and immune status. Many investigations have reported that CONUT scores are independent disease-free survival (DFS) and OS predictors of malignant tumors (7–9, 30); 60-month DFS and OS rates in patients with cervical cancer in a CL group were significantly higher when compared with CH group rates. High CONUT scores were related to lymph node metastasis, periuterine invasion, and a poor nutritional status in cervical cancer patients (7). CONUT scores also impacted OS rates in patients with malignant tumors in small cell lung, liver, breast, gastric, renal cell, and colorectal cancers (8, 9, 11, 30–32). However, no studies have investigated correlations between CONUT scores and EC. To address this, we observed that preoperative CONUT scores were closely associated with OS rates in EC, in particular LTS rates at > 60 months after surgery, while no significant differences were identified between CH and CL groups in terms of OS rates at 60 months after surgery. Also, CONUT scores were significantly correlated with other inflammatory and nutritional indicators (NLR, PLR, LMR, and PNI), Multivariate analysis also confirmed the predictive value of PNI, PLR, and LMR for EC survival.

Based on our data, we believe that preoperative CONUT scores may contribute to risk stratification and personalized treatments in EC. When preoperative CONUT scores were high, poor tumor differentiation (G2 and G3) and deep myometrial invasion (>1/2 depth) outcomes were more common, and poor OS rates (low 95.62% vs. high 91.52%, P<0.001) were identified. We recommend that patients with high preoperative CONUT scores should receive more aggressive adjuvant treatment after surgery, and closer follow-up. Our study had some limitations. Firstly, CONUT, PNI, NLR, PLR, and LMR indicators were grouped based on optimal cut-off levels; however, critical indicator ranges across investigations are different, with no unified optimal standard values. Secondly, ours was a retrospective study, therefore some selection bias may have occurred, and thirdly, the study was conducted at a single center. Therefore, future larger-scale prospective multicenter studies are warranted to confirm our results.

## Conclusions

In EC patients, high CONUT scores were associated with poor clinical prognoses. Patients with CONUT scores ≥1 had a 2.14 times higher risk of death when compared with patients with CONUT scores <1. CONUT scores were related to tumor differentiation and muscle invasion depth in EC. No significant differences in OS rates were identified between CH and CL groups over 60 months, and LTS rates after >60 months were significantly lower in CH patients when compared with CL patients, especially for those with type II disease.

## Data availability statement

The raw data supporting the conclusions of this article will be made available by the authors, without undue reservation.

## Ethics statement

The studies involving human participants were reviewed and approved by Ethics Committee of Beijing Obstetrics and Gynecology Hospital, Capital Medical University. The patients/participants provided their written informed consent to participate in this study.

## Author contributions

Establishment of database: JY, QW, JC, JW, and YZ. Drafting manuscript: JY, QW, and YZ. Statistical analysis: JY, and QW. Preparing the figures: JY. Editing and revising the manuscript: YZ. All authors contributed to the article and approved the submitted version.
